# System based practice: a concept analysis

**Published:** 2016-04

**Authors:** SHAHRAM YAZDANI, FAKHROLSADAT HOSSEINI, SOLEIMAN AHMADY

**Affiliations:** 1 School of Medical Education Sciences, Shahid Beheshti University of Medical Sciences, Tehran, Iran;; 2 School of Medical Sciences, Shahid Beheshti University of Medical Sciences, Tehran, Iran;; 3 School of Medical Education Sciences, Shahid Beheshti University of Medical Sciences, Tehran, Iran

**Keywords:** Accreditation, Medical education, Competency-based education, Concept

## Abstract

**Introduction:**

Systems-Based Practice (SBP) is one of the six competencies introduced by the ACGME for physicians to provide high quality of care and also the most challenging of them in performance, training, and evaluation of medical students. This concept analysis clarifies the concept of SBP by identifying its components to make it possible to differentiate it from other similar concepts. For proper training of SBP and to ensure these competencies in physicians, it is necessary to have an operational definition, and SBP’s components must be precisely defined in order to provide valid and reliable assessment tools.

**Methods:**

Walker & Avant’s approach to concept analysis was performed in eight stages: choosing a concept, determining the purpose of analysis, identifying all uses of the concept, defining attributes, identifying a model case, identifying borderline, related, and contrary cases, identifying antecedents and consequences, and defining empirical referents.

**Results:**

Based on the analysis undertaken, the attributes of SBP includes knowledge of the system, balanced decision between patients’ need and system goals, effective role playing in interprofessional health care team, system level of health advocacy, and acting for system improvement. System thinking and a functional system are antecedents and system goals are consequences. A case model, as well as border, and contrary cases of SBP, has been introduced.

**Conclusion:**

he identification of SBP attributes in this study contributes to the body of knowledge in SBP and reduces the ambiguity of this concept to make it possible for applying it in training of different medical specialties. Also, it would be possible to develop and use more precise tools to evaluate SBP competency by using empirical referents of the analysis.

## Introduction

Since 1998, The Accreditation Council for Graduate Medical Education (ACGME) and American Board of Medical Specialties (AMBS) have defined 6 essential competencies for physicians to provide high quality of care including patient care, medical knowledge, practice-based learning and improvement (PBLI), communication skills, professionalism, and system based practice (SBP) (1).Green et al. (2) maintain that this process has affected the accreditation in USA since July 2002 and this paradigm shift hailed as the Flexnerian revolution of the 21^st^century, is aimed to enhance the ability of physicians to verify who are competent, at a minimum, to deliver safe and effective patient care (3). Competency-based education and these competencies have been used at all levels of medical education from undergraduate, residency, and continuing medical education and its importance has been emphasized (4). Although an understanding of systems is essential to improve the quality and safety of patient care, there is a lack of literature about how to integrate SBP and systems thinking into medical education (5). The SBP competency is challenging concerning definition, performance, training, and evaluation (5-7) and even are considered the most challenging among the six competencies of ACGM (8).When ACGME developed these competencies, it used “minimal language”(Box1) (9) on purpose to enable its flexibility in each program when including competencies (10).

**Box 1:** ACGM’s definition of SBP**

*“Residents must demonstrate an awareness of and responsiveness to the larger context and system of health care, as well as the ability to call effectively on other resources in the system to provide optimal health care.* *Residents are expected to:* *Work effectively in various health care delivery settings and systems relevant to their clinical specialty* *Coordinate patient care within the health care system relevant to their clinical specialty* *Incorporate considerations of cost awareness and risk-benefit analysis in patient and/or population-based care as appropriate* *Advocate for quality patient care and optimal patient care systems* *Work in interprofessional teams to enhance patient safety and improve patient care quality; and* *Participate in identifying system errors and implementing potential systems solutions.”*

ACGME has not yet specifically defined the knowledge and skill of SBP (11). SBP scope is not well identified (12) and these definitions need to be expanded and more explained (13).Graham considers the necessity of this type of definition in using multiple type programs and also has mentioned that this general definition is ambiguous and does not show clearly what physicians need to demonstrate in the SBP competency (14). Without a common understanding of the SBP competency and appropriate methods of assessment consistent with the competency, educators cannot hope to effectively incorporate SBP into daily practice of physicians (15). The aim of this study is to identify SBP’s components by concept analysis for using it in practice and training in different medical education fields and provide a basis for valid and reliable evaluation tools of the competency.

## Methods

Concept analysis is a formal and rigorous process by which an abstract concept is explored, made transparent, defined, and differentiated from similar concepts to be used in theory formulation and communication about it (16, 17). By breaking a concept to its components, its explicability increases and creates a basis for operational definitions (16).To start an assessment tool, items can be created by using empirical referentsthat reflect each defined attribute (16). Xyrichis and Ream (18) have mentioned various approaches for concept analysis including methods of Rogders (19), Walker and Avant (16), Morse (20), and Meleis (21), but Walker and Avant’s method is the most commonly used. We used Walker and Avant’s method with eight steps (16) including choosing a concept, determining  the aims of analysis, identifying the uses of the concept, determining the defining attributes, identifying model case, additional cases(borderline, related, and contrary cases), antecedents and consequences, and  defining empirical referents. As described in the introduction, this concept was important and we needed an operational definition as a basis for teaching and assessment of SBP. We searched the uses of the concept in literature. In this study, only scientific articles have been used as a resource; for this purpose we conducted a search strategy in Medline and ERIC without any time restriction (till April 2015):("System-based practice" OR "systems-based practice") AND (ACGME OR "Accreditation Council for Graduate Medical Education").The resulting literature was initially screened by reviewing titles and abstracts for relevance. 127 articles were extracted in the initial search; 16 articles were identified irrelevant (some themes like work hour, etc.) and the rest were studied regarding the concept of SBP. Regarding relevance, related articles were also extracted based on the references (ancestry searching) and170 articles were included totally. Determining defining attributes is the heart of concept analysis. It has been tried to show the collection’s attributes that are most relevant to the concept and give the highest knowledge to the analyzer. Searching resources at this stage helped the final choice of defined attributes and provided evidence for analysis. Also, the review was used for finding real examples, developing model case, borderline and related cases, and determining empirical referents. Those were constructed by authors. Antecedents are events or incidents that  must occur prior to the occurrence of the concept and consequences are, on the contrary, events that occurs as a result of the occurrence of the concept. Empirical referents are classes or categories of actual phenomena that by their existence or presence demonstrate the occurrence of the concept itself (16). SBP’s empirical referents were derived from the articles.

## Results

**Identifying the uses of the concept**

In the review, 3 articles were found that specifically had worked on SBP components (11, 14, 22). In Graham’s study (14) three major resident roles for SBP emerged through content analysis of 14 nominal groups of stakeholders: resident as Self-Manager, Team Collaborator, and Patient Advocate. Some concepts emphasized in the ACGME definition like using cost–benefit analysis were conspicuously absent from the healthcare team generated list. It showed that there are gaps between the key stakeholders prioritizations about the ACGME definition of SBP. Graham, et al. in a second study (11) developed a taxonomy and defined six general resident roles: system consultant, care coordinator, resource manager, patient advocate, team collaborator, and system evaluator. Ranz, et al. (22) conducted an exploratory study aimed at developing a conceptual model for SBP in psychiatry. In this study, principal-components analysis indicated that SBP could best be represented by 17 items, in four factors, defined here as Team Member, Information Integrator, Resource Manager, and Patient Care Advocate. Of course, they considered Graham’s taxonomy as a baseline and believed that the roles presented here differ somewhat from the roles established by Graham, as they capture the specialty-specific aspects of SBP as it applies to psychiatry. Aside from these articles, various quotes from ACGME definition have been cited in the literature which have some similarities and discrepancies (4, 7, 23-28). Working in various health care settings, coordination of care within system, cost awareness and risk–benefit analysis, patient advocacy, working in interprofessional teams for enhancing patient safety and endeavor to solve system errors have frequently been mentioned (7, 24-26,28).

**Defining attributes**

There were some differences in definitions in the articles. We considered these similarities and discrepancies and tried to define attributes of SBP comprehensively and in a flexible way.

1. Knowledge of the system 

2. Balanced decision between patient needs and system goals.

3. Effective role playing in interprofessional team 

4 .Health advocacy at system level

5. Acting for system improvement

**Knowledge of the system**

Knowledge of the system is one of the defining attributes of SBP. Importance of knowledge of system and knowing how types of medical practice and delivery systems differ from one another, including priorities, restrictions and their effects on medical practice, have been emphasized in some literature (23-26). It is logical when someone does not have detailed information about the system, (s)he cannot perform an effective practice or interaction within the system and cannot allocate resources appropriately.

**Balanced decision between patient needs and system goals**

Health care cost effectiveness and allocating resources have been mentioned in all definitions of SBP in the literature with emphasis on not compromising patient care (7-9,24-26,28) which represent the balance in decision making. Each practice is based on decisions but system based decisions have different bases. It should be balanced between the patient needs and system goals. This point is the difference between SBP and traditional practice, in which disease and patients determine direction of the decisions. This balance is achieved base on system resources, system limitation/restraints/constraints, and system priorities.

**Effective role playing in interprofessional health care team**

Central point in SBP is assisting the patients in dealing with complexities of the system and communicationis the key point (13). The care takes shape in interprofessional health care team and   appropriate role playing of all team members affect the quality of patient care. Working in team has been mentioned in several definitions of SBP (4, 7-9,26, 28). A physician can have different roles in health care team including: communicator (11, 14, 22), collaborator (11, 22), consultant (11, 28), coordinator (11, 22), etc.

**Health advocacy at system level**

Patient advocacy is one of the elements included in the definition of SBP by ACGME. While physician’s advocacy means evolving (29), Earnest, et al. (30) define patient’s support as: "Action by a physician to promote those social, economic, educational, and political changes that ameliorate the suffering and threats to human health and well-being that he or she identifies through his or her professional work and expertise." While Earnest, et al. specifically propose political support, Furrow (31) considers a range of legal, administrative, clinical, and patient-centered supports. As advocacy is intrinsic to policymaking, the current crisis in health care suggests that new strategies for improvingthe quality and broadening the scope of health professions’ advocacy are needed (29). Feldman believes “to remain a profession of scientists and clinicians, we must have an open interchange with the public and with our elected leaders.” We recommend the term “health advocacy” as in ACGME definition has mentioned “population-based care “(9) and in some cases in the literature, problems of SBP projects were about community and population. We can suppose intra- and extra-system levels for health advocacy, but only the intra-system (system level) scope is mentioned here, as extra-system level cannot cover the system definition. Here is an ambiguity about “community leadership”, which has been cited in physicians’ responsibilities in many resources. Community leadership overlaps SBP competency, but SBP cannot cover extra-system behaviors and maybe this is the point neglected in ACGME competencies.

**Acting for system improvement**

**Case model**

Walker and Avant define case model in this way: “A model case is an example of the use of the concept that demonstrates all the defining attributes of the concept. That is, the model case should be a pure case of the concept, a paradigmatic example, or a pure exemplar”(16). The constructed case model for SBP has been shown in here:

“*A 56-year-old woman with previous diagnosis of severe arthrosis refers to her family physician with severe knee pain. She hasn’t been able to go upstairs since six months ago and is dependent on her children for housework and it saddens her. The physician prescribes glucosamine, chondroitin, and analgesic, according to the national guidelines and explains that she should take these drugs for at least one year. She has been informed about new technical developments of artificial knees and wants to know if she can have joint replacement. The physician explains according to the national guidelines, her disease usually becomes controlled with these drugs for one year and so unnecessary surgery should be avoided. He explains further that as far as the disease burden of arthrosis increases with aging (increasing life expectancy), there is possibility of unnecessary queue in the shorter time and the national guidelines do not recommend joint replacement before one and a half year. The physician convinces her that the technologies used in the artificial joints will improve over time and joint replacement after one and a half year is fruitful for both patients and the system. She takes her prescription and leaves while expressing fear from the pain and disability which make her more dependent on her children during this time. After that the physician thinks about a few patients visited last month with this complaint and they were unable to tolerate pain despite using strong analgesic. She has concern about impact of the decision on patients’ daily life and putting them at risk if their symptoms wouldn’t relieve adequately. She thinks the time of one and a half year must be revised, but he doesn’t find strong evidence about it in the literature. She extracts and classifies data from the patients’ records and reports them to scientific association responsible for developing the guideline. She mentions that the findings may not be enough for conclusion, but it is necessary to consider it as a serious issue and declares her willingness to participate in the project if it is funded. The scientific association has received similar information and based on them orders a review on this section of the guideline and announces physicians’ reporters”.*

In the case model, the physician is well aware of the clinical guideline (knowledge of the system) and has done his duties as a system collaborator (effective role playing). He also plays her role as a system consultant for the patient, explains necessity of the balance between the patient needs and the system in a logical manner and practiced based on a balanced decision (balanced decision between patient needs and system goals). He also does not stop and considers the impact of such a decision on patients and reflects his criticism on behalf of patient (health advocacy) and participates in the system change (acting for system improvement).

**Additional cases**

Borderline, related, and contrary cases complete justification about the defining attributes. Borderline cases are examples that contain most of the defining attributes of the concept, but not all of them. Related cases do not include all attributes and they are similar to the case model but are not them. Contrary cases are clear instances of “not the concept”(16).Here constructed contrary and borderline cases for SBP made by authors are shown.

**Contrary case**

"*A 56-year-old woman with a previous diagnosis of severe arthritis refers to her family physician with severe knee pain. She hasn’t been able to go upstairs since six months ago and is dependent on her children for housework and it saddens her. The physician informed her about new artificial joints, according to the newest articles. He explained that her pain could be controlled by glucosamine, chondroitin, and analgesic but joint replacement must be performed ultimately. By considering patient’s dependency on others, the physician refers her to an orthopedist and he taught it might be better to perform this sooner."*

**Borderline case**

“*A 70-year-old man, with a previous diagnosis of severe arthritis of the right knee, refers to his family physician and complains about severity of pain in the last month. He hasn’t used medication and the physician prescribes glucosamine, chondroitin, and analgesic, according to the national guidelines and explains that he should take these drugs for at least one and half year. The patient asked about the difference between foreign and domestic medications. The physician explains to him that the effectiveness of both is the same, but foreign drugs impose unnecessary costs to insurance companies. The patient believes that he has paid insurance premium for such days and insists on prescribing foreign drugs. Just patient’s satisfaction isn’t a good reason for prescribing foreign drugs and the clinical guidelines don’t support it.Although the physician knows that imposing unnecessary costs on the insurance company means health system would lose its resources for critical incidences, he prescribes the foreign analgesic only to increase patient satisfaction”.*

In the contrary case, although the physician informs the patient as an informed consultant, no systemic approach is observed in his advice and his view is only from professional perspective. If the physician had an evidence-based approach in this case and had criticized accuracy and importance of the articles, it would also have been evidence-based practice, but absolutely not SBP, as the system concerns were not considered in it.

In borderline case, the physician has a good knowledge and has informed his patient about the perspective’s system as an informed consultant. But the final decision was not a balanced decision between the patient needs and the system and therefore the system lost its resources. This case has some attributes of SBP but finally could be considered as a patient-centered practice and not SBP.

**Related cases**

The concept of SBP is related to other general competencies and difficult to separate (42). This concept has been frequently used with PBLI in the literatures. In clinical settings, we can operationalize these concepts by asking two separate questions:

1. The PBLI question: “How can I improve the care formy patients? (15) Or “How can I change my own practice to improve patient care”? (43)

2. The SBP question: “How can I improve  the system of care?(15) Or “How can I work with the health care system to improve patient care”? (43)

PBLI improves care in a private practice while SBP needs understanding and improving care within the health care system (41). In both, we have “acting for improvement but in different scopes.” PBL is improvement in individual practice, but SBP is about the system.

Apart from PBLI, evidence-based practice or ethical-based decision making can be named as related cases in both of which a balanced decision is performed based on evidence or ethics criteria, not on system’s goals. Of course, it can be possible to apply evidence or ethics criteria simultaneously with system’s criteria, in which the system decision would be based on ethics and/or evidence but we don’t recommend to integrate them because there are systems which aren’t evidence-based or have ethical challenges and therefore it would be better to see them separately.

**Antecedents and consequences**

SBP’s antecedents in this study determined systems thinking and existence of a functioning system. On the other hand, SBP’s consequences are system’s goals. Several definitions of SBP (23-26) have been mentioned to understand how physician’s patient care affects other health care professionals, the health care organization, and the larger society and how these elements of the system affect physician practice in system thinking.

Systems thinking means the focus has moved from the individual to consideration of the function or dysfunction of the system as a whole (44). Systems thinking as an ability to analyze systems as a whole, includes the recognition of essential interrelationships within a system and between the subsystems. Systems thinking is not a basic structure for SBP, but can be considered as a cognitive requirement for SBP behavior (44).

 Another antecedent which is more important is the functioning system. But what is a system, in which practice is performed?

IOM defines system as a group of independent elements (both human and non-human), interacting to each other to achieve a goal (45). Independent systems of health systems include clinics, hospitals, care process, and legal systems that affect the patient care (44).The health care is provided in a complex environment and acts as a complex adaptive system (CAS) operation (46).

SBP’s consequences in this concept analysis are health care goals. IOM defines six specific goals for health systems, including: safe, effective, patient-centered, timely, efficient, and equitable (47). Similar goals have been developed in Australia, Canada, and England with more or less similar issues. In 2005, Bingham, et al. (48) introduced a health care matrix which was a conceptual framework that projects an “episode of care” as the large and complex picture that provides a glimpse into the interaction between quality outcomes (IOM Aims for Improvement) and ACGME core competencies necessary to affect those outcomes to make readily apparent the tight linkage between competencies and outcomes. Lee, et al. used the matrix with some modifications in clinical education and believe their achievements were encouraging (49).

**Empirical referents of SBP**

The final step in a concept analysis is empirical referents.” When a concept analysis is nearing completion, the question arises, "If we are to measure this concept or determine its existence in the real world, how do we do so?" Empirical referents are classes or categories of actual phenomena that by their existence or presence demonstrate the occurrence of the concept itself (16).”Empirical referents of the SBP were determined as follows in Box 2.

**Box 2:** Empirical referents of the SBP

knowledge of the system Demonstrate knowledge of the public structure and private organizations (22). Demonstrate understanding of the financing and regulation of the practice (22). Demonstrate Knowing how to advocate for health promotion and the prevention of disease (22). Demonstrate being aware of costs (11). Demonstrate being familiar with different patient insurances (11, 14). Demonstrates knowledge of resources available for patient (14). Balanced decision between patient needs and system goals System resources Practice cost-effective healthcare and resource allocation (22). Select computed tomography (CT scan) only if needed (11). System limitations/constrains Practice within the constraints of the system (11). System priorities Uses approved protocols in the clinical practice. Effective role playing in interprofessional health care team Communicator Interact with other practitioners such as physicians, therapists, and etc. to communicate about treatment, follow-up plans, and other concerns of patients (14). Communicate well with colleagues (11). Document, in progress notes, the patient’s use of services specified in the treatment plan (22). Prepare an adequate discharge summary and plan (22). Collaborator Work in inter-professional teams including nurses, social workers, and etc. (22). Uses approved protocols in the clinical practice. Coordinator Coordinate patient care within the health care system relevant to their clinical expertise (22). Timely refer of patients to appropriate services (11). Consultant Provide advice and training to other medical occupations associated with the correct use of video sources (28). Discussing the limitations of different insurance plans with patients and their families (14). Health advocacy in the system level Act on behalf of the patient Ensures proper follow-up with/for the patient (11, 14). Assist patients in dealing with system complexities (22). Empowers patients to know about their insurance plan, alternative treatment plans and etc. (11, 14, 22). Empowers patients by providing specific education to patients about diagnosis and treatment plan (14). Transmits effectively needs/wishes of patients and health care providers to other services (14). Act on behalf of the population Increase public awareness about mercury toxicity used in cosmetic creams (34). identify high-risk areas, and risk factors useful in directing preventive community efforts (34). Improve immunization rates of infants, children, and adults in his/her area (34). Action for system improvement System assessment Conduct systematic analysis of the system’s processes (11). Work with healthcare managers and healthcare providers to assess health care (22). Discuss treatment protocols with other physicians, social workers, and medical students to perform better care (11, 14). Participate in identifying system errors (11, 22). System feedback Reports evidence-based benefits and risks for treatment plans (11). Gives structured feedback to the social worker (14). System change Participate in implementing potential systems solutions (22). Work to increase patient’s safety (22). Proposing suggestions for system upgrades (14).

## Discussion

The nature of medical practice in the 21st century has been changed from individual practitioners to interdisciplinary teams of health care providers (50). These new models of health care delivery and care coordination place the physician in a central role that is part of a rich matrix of health care providers (51). To better prepare for this systems-based, interdisciplinary approach to health care delivery and based on this analysis, physicians must know well the system, knowing and playing their role in the team and making decisions based on system resources, limitations and priorities. But this won’t be enough for SBP and acting on behalf of the patient/population and the system will be necessary to complete this concept and achieve quality of care. Bingham, et al. developed a Healthcare Matrix that links the IOM aims for improvement and the six ACGME Core Competencies including SBP. The Matrix projects an episode of care as an interaction between quality outcomes and core competencies necessary to affect those outcomes (48) and in this analysis, those outcomes are considered as the consequences of SBP. If the practice changes, the outcome will also change. Of course, SBP is one of six core competencies and must be integrated to others for maximizing quality.

Practice, training and assessment of SBP require a precise definition of the concept and the analysis clarifies the concept of SBP by introducing itsantecedents, defining attributes, and consequences. The model case, contrary and borderline cases of SPB as typical examples could play an important role in accurate training of SBP. Empirical referents of SBP are means by which we can recognize or measure the defining attributes and are useful in instrumental development. They are observable behavior by which to check the existence of each attribute in medical practice.

“Concept analysis is ultimately only a careful examination and description of a word or term and its uses in the language coupled with an explanation of how it is “like" and "not like" other related words or terms.” Although concept analysis has a precise process, the final product is always tentative, which can be considered as a limitation for the study. In other words, the concepts are not written on stone and have a dynamic nature, changing by the cultural, social, and contextual factors that should be considered when applying it in different fields (16).

## Conclusion

The analysis of the concept of SBP represents a step toward its understanding and implementation in medical education. This concept analysis can be applied in any medical field as a basis for education, evaluation, and instrument development and further studies could measure its universality. The identification of attributes which are critical to the concept, its antecedents and consequences contribute to the body of knowledge in SBP. Moreover, it offers conceptual and operational foundations for teachers and researchers in the medical education. 
